# Identification of key genes underlying radiosensitivity and radioresistance in endometrial cancer through integrated bioinformatics analysis

**DOI:** 10.3389/fgene.2025.1469610

**Published:** 2025-01-24

**Authors:** Chunhui Wan, Lei Zhang, Ting Yu, Hui Lu, Han Xiao, Juan Du

**Affiliations:** ^1^ Precision Medical Center, Wuhan Children’s Hospital (Wuhan Maternal and Child Healthcare Hospital), Tongji Medical College, Huazhong University of Science and Technology, Wuhan, China; ^2^ Institute of Maternal and Child Health, Wuhan Children’s Hospital (Wuhan Maternal and Child Healthcare Hospital), Tongji Medical College, Huazhong University of Science and Technology, Wuhan, Hubei, China

**Keywords:** endometrial cancer (UCEC), radiation therapy, radiosensitivity, radioresistance, bioinformatics analysis

## Abstract

**Introduction:**

Radiation therapy is crucial in the treatment of endometrial cancer (UCEC). Patients exhibit significant variability in radiosensitivity, affecting therapeutic effect. Scarcity of studies exploring the gene-radiosensitivity relationship based on clinical data. Underlying molecular mechanisms of radiosensitivity and radioresistance require further investigation.

**Methods:**

Study aimed to reveal molecular mechanisms underlying radiosensitivity and radioresistance in UCEC patients. Included 12 radiosensitive and 20 radioresistant UCEC samples. Conducted differential expression analysis to screen for significantly different genes between groups. Applied Lasso regression and randomized survival forest model to identify key genes. Performed functional annotation, correlation analysis, and survival analysis on key genes.

**Results:**

Key genes positively correlated with UCEC tumorigenesis-related genes in the radioresistant group. Reduction in the proportion of Macrophages.M0 observed in the radioresistant group, associated with poor prognosis. GO and GSVA analyses revealed biological processes and signaling pathways involved in key genes. High expression of MARCKS, MACC1, and GRB10 correlated with poorer survival rates. High expression of NINJ2 correlated with higher survival rates and higher sensitivity to radiation therapy.

**Discussion:**

Study contributes to a deeper understanding of UCEC radiosensitivity. Provides theoretical support for the development of personalized radiotherapy regimens in clinical practice. Potential to improve prognosis and quality of life of patients.

## 1 Introduction

Uterine Corpus Endometrial Carcinoma (UCEC) is a malignant epithelial tumor originating from the endometrium, which is one of the common gynecological tumors. It is estimated that approximately 76,000 women worldwide die from UCEC each year (Urick and Bell, 2019). Based on the surgical staging, postoperative adjuvant treatment options typically include vaginal brachytherapy or adjuvant systemic therapy (Abu-Rustum et al., 2023). The efficacy of radiotherapy in local control of UCEC is well-established (Blake et al., 2009). However, regional and distal metastases remain significant factors affecting 5-year survival (National Cancer Institute, 2023). To address this issue, it is crucial to identify biomarkers that can accurately, sensitively, and specifically predict the sensitivity of UCEC to radiotherapy. These biomarkers can help to improve the effectiveness of radiotherapy and, consequently, enhance the 5-year survival rate of patients.

Radiotherapy primarily operates by disrupting DNA double-strand breaks (DSBs) or free radicals and reactive oxygen species indirectly induced by ionizing radiation in cancer cells, resulting in cancer cell death or severe damage (Zhao et al., 2023). When developing a radiotherapy regimen, it is crucial to select a radiotherapy dose that is effective in destroying tumor cells while minimizing damage to normal cells (Pavlopoulou et al., 2017). However, individual differences can lead to some tumor cells being less sensitive to the effective dose of radiotherapy, resulting in cancer recurrence and metastasis, and consequently reducing the survival rate post-radiotherapy (Kim et al., 2015). Numerous factors influence radiosensitivity, with tumor proliferation dynamics, clonal cell number, degree of hypoxia, and intrinsic radiosensitivity typically considered the main contributors (Hennequin et al., 2008). Among these factors, specific genes or gene products are closely associated with radiosensitivity. For example, cell division cycle genes involved in cell cycle regulation modulate the response of triple-negative breast cancer cells to radiotherapy (Han and Fu, 2015). Additionally, genes encoding apoptotic proteins, such as BCL2 and BCL2L1, which regulate apoptosis, can also impact radiosensitivity (Pavlopoulou et al., 2017). In a clinical study on rectal cancer, upregulation of the PLAGL2, ZNF337 and ALG10 genes was associated with radiosensitivity (Zhao et al., 2023). Similarly, in clinical data from breast cancer patients, the expression of TPD52 and NFKB1 genes correlated with radiosensitivity (Sims et al., 2009). Furthermore, carriers of BRCA1, BRCA2, and PALB2 genes exhibit heightened sensitivity to radiation, necessitating particular caution when administering radiotherapy to these individuals (Chadwick, 2023). However, research on the genetic determinants of sensitivity and specificity in UCEC radiotherapy remains inadequate.

During radiation therapy for UCEC, the ability to identify these key molecular genetic features in advance would significantly facilitate the development of more precise radiotherapy strategies. This method enhances the precision of radiotherapy targeting endometrial tumor cells, reduces harm to healthy cell. To achieve this, the present study was dedicated to an in-depth analysis of a database of UCEC patients who exhibited significant differences in survival outcomes after radiotherapy, specifically focusing on comparing and extracting information about differentially expressed genes. High expression of radiosensitive genes suggests radiotherapy as the primary treatment, while high expression of radioresistant genes indicates the need for adjuvant therapies. Our objective is to screen for specific biomarkers that are closely related to radiotherapy sensitivity, which will serve as a crucial foundation for optimizing individualized radiotherapy regimens. Through this endeavor, we anticipate providing UCEC patients with more personalized and targeted radiotherapy strategies, effectively reducing mortality and enhancing treatment outcomes.

## 2 Materials and methods

### 2.1 Data collection and screening

We obtained the clinical data on UCEC patients post-radiotherapy from The Cancer Genome Atlas Program (TCGA) database, totaling 557 cases. The data were updated to January 10, 2024. After rigorous screening, we excluded samples lost to follow-up, those with mismatched clinical samples and sequencing data, and duplicates, ultimately identifying 32 valid samples. These samples were divided into two groups based on patient survival post-radiotherapy: the radiotherapy-resistant group (survival within 5 years), containing 20 samples; and the radiotherapy-sensitive group (survival over 5 years), containing 12 samples.

### 2.2 Analysis of differentially expressed genes (DEGs)

Using the DEseq2 package in R, we calculated gene counts and screened for different genes with log2Foldchange greater than 1 or less than −1 and P-value less than 0.01. These genes were visualized using volcano plots and heatmaps to illustrate differences in gene expression levels.

### 2.3 Screening and validation of core genes

We employed the glmnet package in R for Lasso analysis, combined with Cox analysis, to screen for genes with prognostic value. Subsequently, these prognostic genes were ranked by importance using randomized survival forest analysis. Genes with p-cutoff less than 0.02 for downscaling and importance score greater than 0.3 were defined as core genes.

### 2.4 Study of the relationship between core genes and disease-associated genes

We sourced 24 disease genes associated with UCEC tumorigenesis from the GeneCards database (https://www.genecards.org/). We determined the expression of these disease-related genes in both groups and assessed their significance and correlation with core genes using the Pearson coefficient.

### 2.5 Immune infiltration analysis

We conducted a deep analysis of RNA-seq data from the radiation response and non-response groups using the CIBERSORT algorithm. We identified 22 immune cell types associated with radiotherapy prognosis. Next, we performed a comparative analysis of immune cells in these two groups and calculated the Pearson correlation between immune cells within each patient.

### 2.6 Functional enrichment of differentially expressed genes (DEGs)

We performed Gene Ontology (GO) functional annotation and Kyoto Encyclopedia of Genes and Genomes (KEGG) signaling pathway enrichment analysis on the screened DEGs using the DAVID database. This step allowed us to understand the roles of these genes in biological processes and the signaling pathways involved. We screened for signaling pathways with P-value less than 0.05.

Additionally, we conducted Gene Set Variation Analysis (GSVA) was performed using the GSVA R package (v1.34.0 and Gene Set Enrichment Analysis (GSEA) using the ClusterProfiler R package (v3.14.3) with the database reference c2.all.v2023.2.Hs.entrez.gmt under version msigdb_v2023.2.Hs_GMTs.

### 2.7 Survival rate comparison among two groups

We used the R package survival (v3.2-13) and survminer (v0.4.9) to plot Kaplan-Meier survival curves. Cox proportional hazard models were constructed utilizing the “coxph” function from the survival package and visualized using the “ggforest” function from survminer.

### 2.8 Risk factor linkage maps

Risk factor linkage maps consist of risk score scatterplots, patient life and death scatterplot, and gene expression heatmap. These maps visualize the relationship between risk scores, expression levels, and patient survival rates of risk factors.

We analyzed survival time and gene expression levels (FPKM) of five risk genes in patients using ggrisk package (v1.3).

### 2.9 Prognostic alignment diagram

The estimation and prediction methods of Regression Modeling Strategies (rms, v6.2-0) are based on Bayesian Partial Proportional Odds Ratio Models for statistical modeling and prediction. The survival method of the object created by the cph function returns an S-function that is used to calculate an estimate of the survival function. We utilized the cph survival analysis model to generate a nomogram illustrating various survival nodes. For plotting the prognostic columns, we selected age, figo_stage, primary_diagnosis (preliminary diagnosis), and the expression levels of the genes GRB10, NINJ2, MACC1, and MARCKS as key indicators.

### 2.10 Validation of key genes using the CPTAC database

To validate the reliability of the TCGA dataset, we chose the CPTAC dataset for further verification. After applying PCA dimensionality reduction analysis to eliminate data with significant dispersion, we observed that selecting survival times greater than 5 years resulted in only 2 radiosensitive cases. Therefore, we adjusted our criterion and selected a survival time greater than 1800 days (approximately 4.92 years) as indicative of radiosensitivity, yielding a total of 6 materials and 8 sequences. Conversely, we defined survival times of 1800 days or less as radioresistant, resulting in 7 materials and 10 sequences. We then validated gene expression and survival data for the four genes selected by TCGA as having a significant effect on survival analysis.

## 3 Results

### 3.1 DEGs analysis and functional enrichment of radiosensitive and radioresistant samples

In the TCGA database, we screened 32 UCEC samples, of which 12 were radiosensitive samples (responders) and 20 were radioresistant samples (non-responders). These samples were then analyzed using the DEseq2 software package to detect DEGs. Based on the screening criteria of a p-value less than 0.01 and log2Foldchange greater than 1, we identified a total of 765 genes with significant differences in expression between radiotherapy responders and non-responders. Among them, 604 genes were upregulated, while 161 genes were downregulated.

**FIGURE 1:**
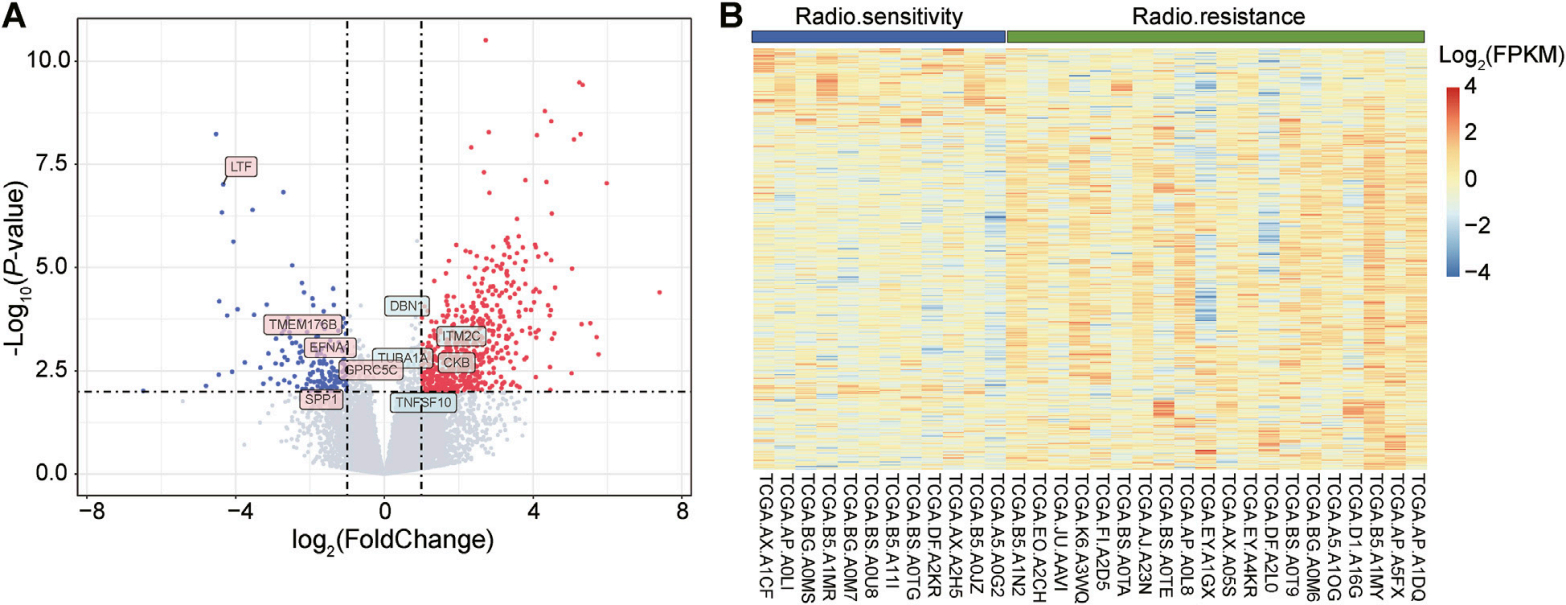
Visual display of differentially expressed genes (DEGs), including volcano and heat maps. **(A)**: The volcano plot depicts the distribution of DEGs. The red dots represent upregulated genes, i.e., genes with increased expression levels, while the green dots represent downregulated genes, i.e., genes with decreased expression levels. **(B)**: Heatmap showing the expression of DEGs in 12 radiosensitive samples (blue) versus 20 radioresistant samples (green). In this heatmap, blue color indicates genes with lower expression levels, while red color indicates genes with higher expression levels.

To visualize the expression patterns of these up- and downregulated genes, we generated a volcano diagram (Figure 1A), which clearly depicts the expression changes of each gene. Additionally, we presented the expression patterns of these DEGs across the 32 samples using heatmaps (Figure 1B). In the heatmap, the first 12 samples belonged to the radiosensitive group (responders), while the last 20 samples belonged to the radioresistant group (non-responders). By comparing the heatmaps of the two groups, we observed a clear difference in the expression of DEGs between them.

### 3.2 Analysis of lasso regression model and random survival forest model of DEGs and identification of key genes

**FIGURE 2:**
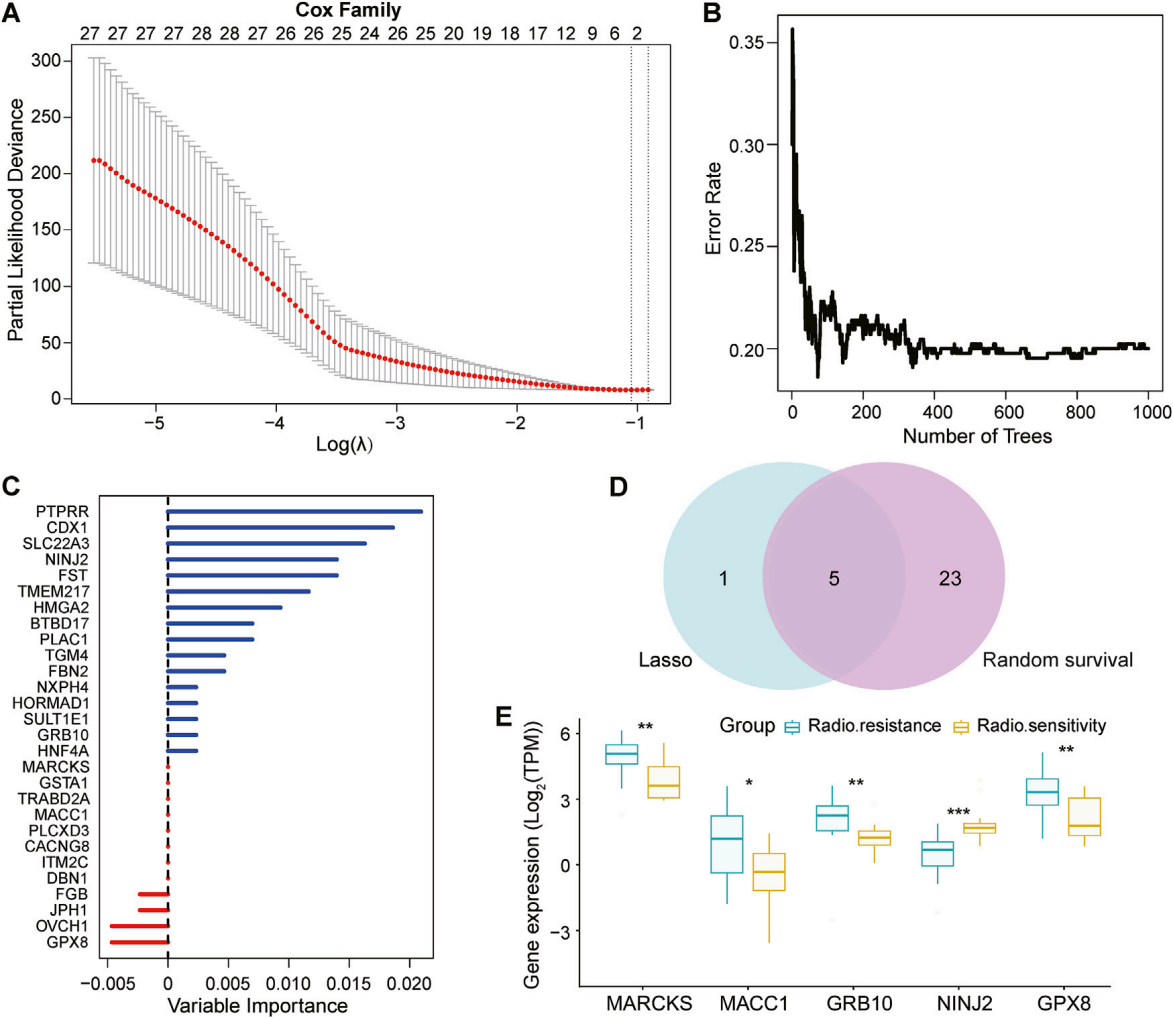
Screening and validation of prognostically relevant key genes. **(A)**: Breast cancer (BRCA) and ovarian cancer (OV) were selected as references, and 162 genes consistent with the trend of 765 DEGs in UCEC were identified by Deseq2 analysis. These 162 genes were screened using the Lasso regression model (based on least squares and penalty coefficients), and six genes with significant predictive value were successfully identified with coefficients that were not 0. **(B, C)**: The same 162 genes were independently analyzed to validate key genes associated with prognosis using the random ForestSRC model, and the model ultimately identified 28 prognostically relevant genes. **(D)**: Wayne plots show that the Lasso regression model and random ForestSRC model together identified 5 common genes that were considered to be prognostically relevant key genes. **(E)**: Box plots showing the 5 genes in common (MARCKS, MACC1, GRB10, NINJ2, GPX8) in the 12 radiosensitive and 20 radioresistant samples.

Our study focused on clinical sample data from patients with UCEC after radiotherapy. The difficulty in obtaining these samples resulted in a relatively limited number of eligible samples, was relatively limited, posing a challenge to the stability and reliability of our results. Furthermore, breast cancer (BRCA), ovarian cancer (OV), and UCEC share complex biological links and several common features (Pane et al., 2020). The development of these tumors is often closely related to similar genetic, environmental, or lifestyle factors (Freuer et al., 2021; Cao et al., 2024). Therefore, we compared the DEGs of BRCA and OV with those of UCEC to gain insights into common genetic alterations driving the progression of these tumors. By comparing their DEGs with those of UCEC, we screened the genes that exhibited similar expression trends to the 765 key genes in UCEC. After rigorous screening, we targeted 162 shared DEGs. Next, we performed an in-depth analysis of these 162 DEGs using the Lasso Cox regression model (Figure 2A). The Lasso regression model achieves variable selection by introducing a penalty term that compresses the coefficients of insignificant variables to zero. By this method, we screened out six genes with non-zero coefficients and significant predictive significance.

Simultaneously, we constructed the random Forest SRC model (Figures 2B, C), an integrated learning method based on decision trees for predicting survival time. With this model, we screened 28 genes associated with UCEC prognosis, which are shown as vertical coordinates in Figure 2C.

To identify the genes jointly identified by both analysis methods, we plotted a Wayne diagram (Figure 2D) showing the intersection of the genes screened by the Lasso regression model and the random survival forest model. The results showed that five genes-MARCKS, MACC1, GRB10, NINJ2, and GPX8-were identified as key genes in both methods.

To further validate the expression differences of these five key genes in UCEC analyzed by the Lasso Cox regression model, we plotted box-and-line plots (Figure 2E) showing their expression in the radiosensitive and radioresistant groups. Specifically, MARCKS, MACC1, GRB10 and GPX8 were identified as radioresistant genes, exhibiting high expression in the radioresistant group. In contrast, NINJ2, as a radiosensitive gene, showed higher expression in the radiosensitive group. This suggests that high expression levels of MARCKS, MACC1, GRB10 and GPX8, may indicate ineffectiveness of radiation treatment for UCEC. Conversely, high expression of the NINJ2 gene may predict a better radiotherapy outcome for UCEC.

### 3.3 Study of the relationship between key genes and disease-related genes

**FIGURE 3:**
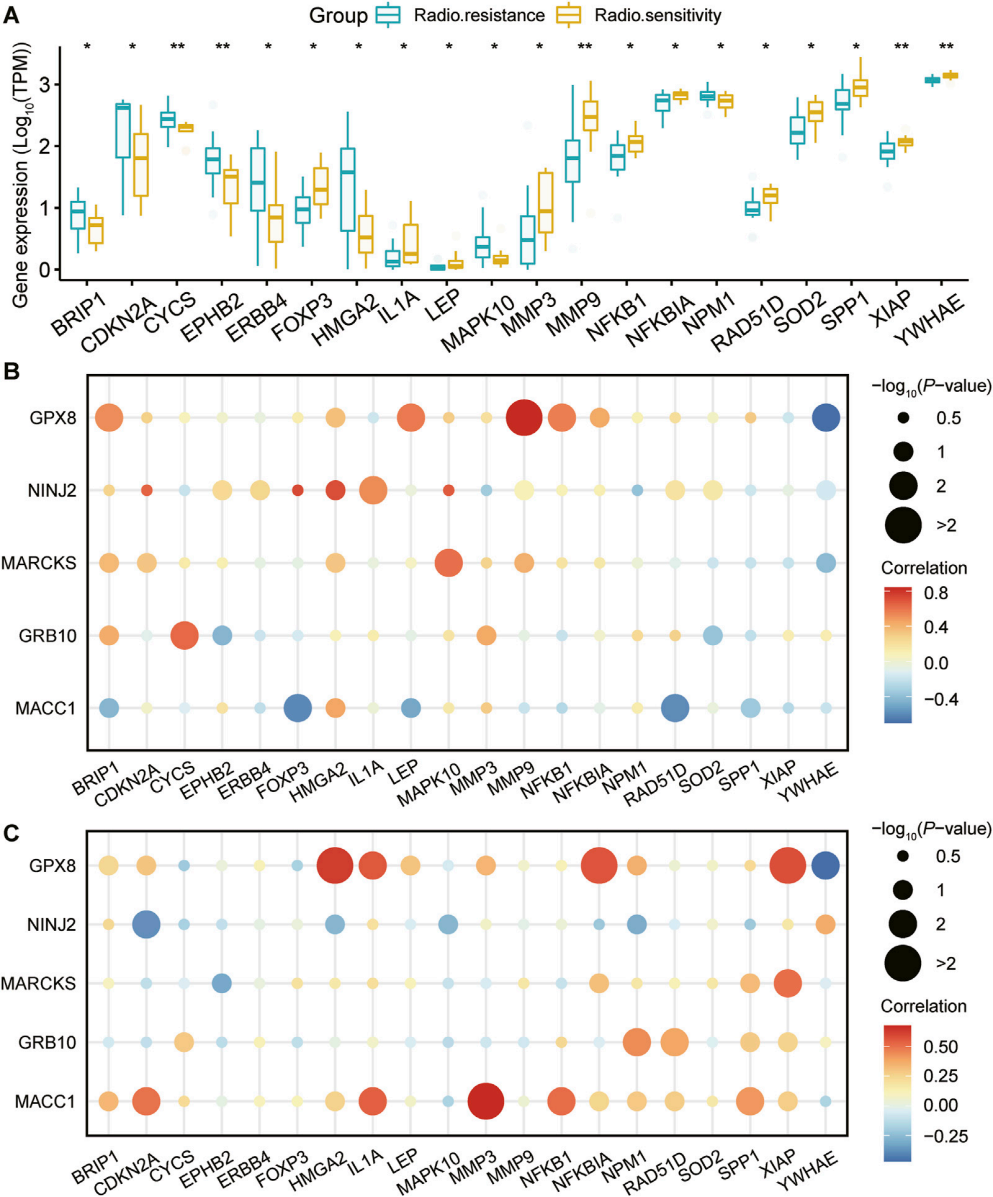
Expression of UCEC-related genes and correlation analysis with prognostic genes. **(A)**: Genes associated with the development of UCEC were retrieved from the GeneCards database (https://www.genecards.org/). Following this, a comprehensive analysis was conducted to evaluate the differential expression of these genes between radiosensitive and radioresistant groups. The figure showcases a list of twenty genes that are associated with the occurrence of UCEC. These genes exhibit significant differences in their expression levels between the radiosensitive and radioresistant groups. Using pearson correlation analysis, the correlation between the five genes strongly associated with prognosis and these 20 UCEC-related genes in different radiation outcomes (**(B)** for radiosensitivity and **(C)** for radioresistance) is visualized. Horizontal coordinate: 20 genes associated with UCEC. Vertical coordinate: 5 genes closely associated with prognosis. Bubble size: -log10PValue, representing the statistical significance of the correlation between genes, the larger the bubble, the higher the significance. Bubble color: reflects the strength of correlation between genes, the darker the red color, the stronger the positive correlation, the darker the blue color, the stronger the negative correlation.

To gain insight into the association between the above five genes related to the prognosis of UCEC and disease occurrence, we first obtained genes linked to UCEC from the Gene Cards database (https://www.genecards.org/). Subsequently, our analysis revealed significant differences in the expression of multiple disease-related genes between the radiosensitive and radiotolerant groups (Figure 3A).

To further explore the relationship between these five key genes and the 20 disease-related genes, we conducted a Pearson correlation analysis. This analysis enabled us to measure the degree of interrelation among these genes at the expression level. The findings are presented in bubble plots, which illustrate the correlations for both the radiosensitive group (Figure 3B) and the radioresistant group (Figure 3C) with respect to the disease-related genes. Notably, the core genes MACC1, GPX8, NINJ2, MARCKS, and GRB10, along with the genes related to UCEC, exhibited significant correlations with the expression of various disease-related genes. Figures 3B, C illustrates that in the radiosensitive group, high NINJ2 expression is positively correlated with CDKN2A, HMGA2, and MAPK10, while in the radioresistant group, this correlation is negative. Similarly, high MACC1 expression shows a positive correlation with IL1A, NFKB1, and SPP1 in the radiosensitive group but a negative one in the radioresistant group. These patterns suggest a strong connection between these genes and the development of radiosensitivity and radiotolerance.

### 3.4 Analysis of the level of immune infiltration in the radiosensitive and radioresistant groups

The tumor microenvironment comprises a complex interplay between tumor cells and their surrounding components, significantly influencing tumor diagnosis, survival outcomes, and therapeutic responsiveness. Tumor immune infiltration occurs when immune cells penetrate the tumor interior. This phenomenon has dual implications: immune cells may exert immunosurveillance to contain tumor progression, while certain immune cells can facilitate tumor escape, thereby accelerating tumor growth. Consequently, there exists a tight correlation between tumor immune infiltration and tumor progression.

**FIGURE 4:**
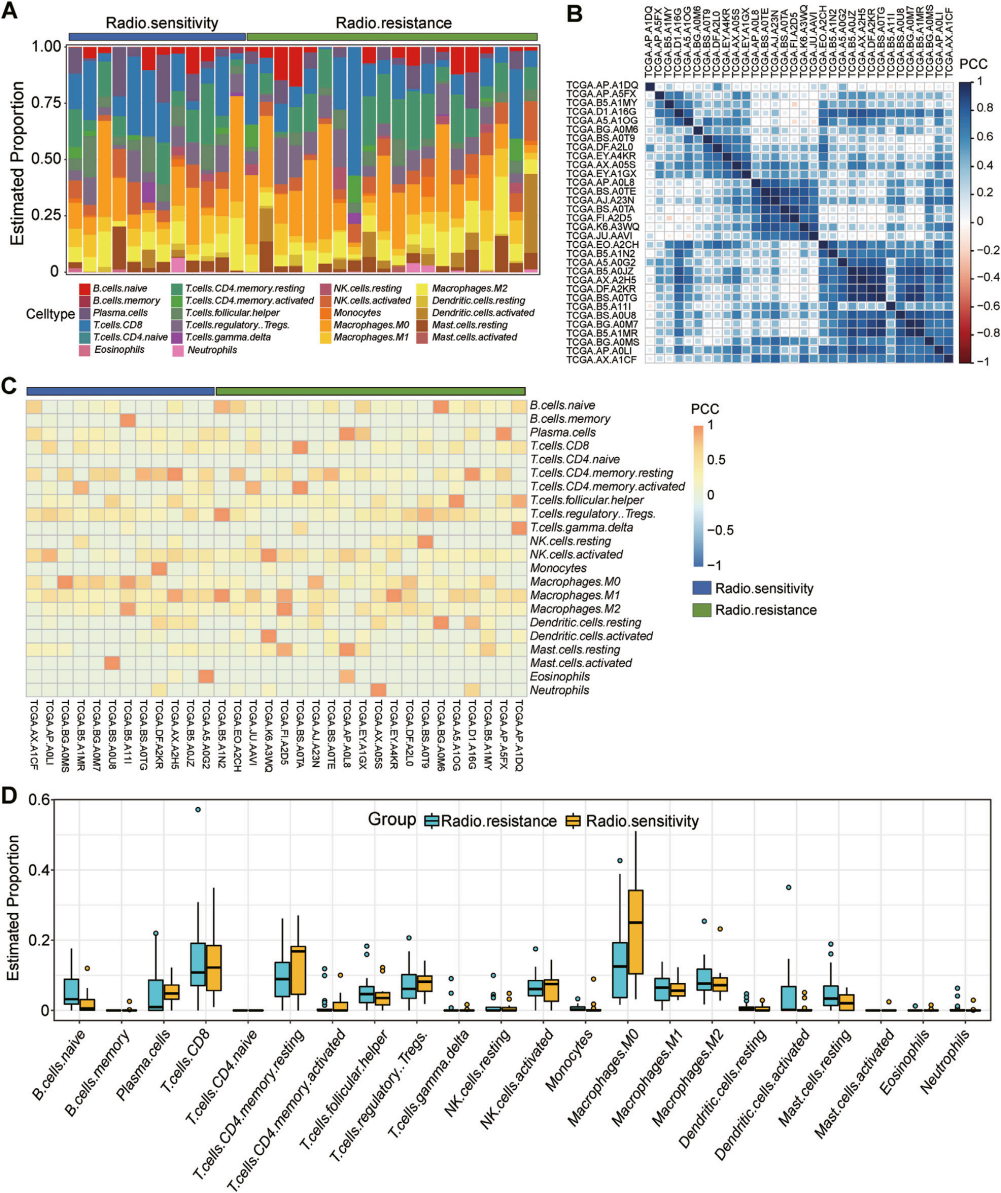
Detailed analysis of immune infiltration in 32 samples. **(A)**: Proportion of immune infiltrating cells in each of the 32 patients *in vivo*, including 12 radiosensitivity (blue) and 20 radioresistance (green). **(B)**: Pearson’s correlation between immune cells in each patient *in vivo*. **(C)**: Expression levels of 22 cell sets associated with immune infiltration in each patient, 12 radiosensitivity (blue) and 20 radioresistance (green). **(D)**: Box plot comparing the difference in expression on immune infiltrating cell sets between the radiosensitive group and the radioresistant group.

To delve deeper into the immune infiltration characteristics of the radiosensitive and radioresistant groups, we performed an immune infiltration analysis using CIBERSORT. Figure 4A clearly shows the proportion of immune-infiltrating cells among the 32 patients, offering visual representation of their immune status. Figure 4B further reveals the Pearson correlation among immune cells within each patient, aiding in the understand the interactions between different immune cells types. For a comprehensive grasp of immune infiltration’s impact on tumor progression, we also analyzed the expression levels of 22 cell sets associated with immune infiltration, as shown in Figure 4C. The expression changes provided deeper insights into the effects of immune infiltration on tumors. Lastly, we compared the expression disparities in immune infiltration cell sets between the radiosensitive and the radioresistant groups using a box-and-line plot (Figure 4D). By integrating the results from Figures 4C, D, we observed that patients in the radioresistant group tended to exhibit lower proportions of macrophage M0 compared to those in the radiosensitive group. However, this difference was not statistically significant, potentially due to our study’s relatively small sample size, which resulted in notably high variability in the results.

### 3.5 GO and GSVA analysis of DEGs and GSEA analysis of key genes

**FIGURE 5:**
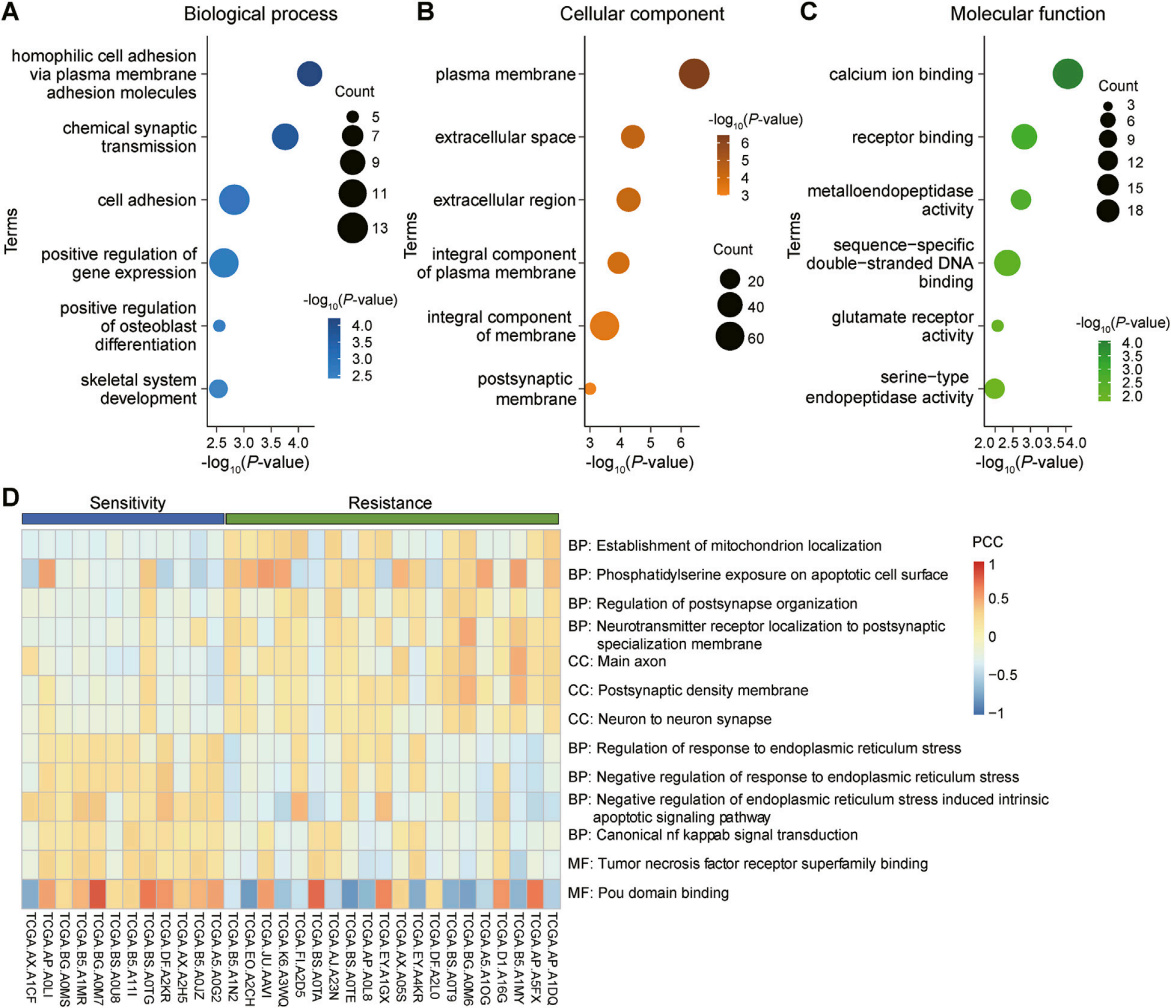
GO analysis and GSVA analysis of differentially expressed genes (DEGs) shared by gynecological tumors. **(A)**: Biological processes in gene ontology (GO) analysis, revealing the potential roles of these genes in tumor biology. **(B)**: Cellular component analysis, detailing the distribution and potential roles of these genes within cells. **(C)**: Molecular functional analysis, revealing the specific functions of these genes at the specific functions at the molecular level. **(D)**: Gene set variation analysis (GSVA) was performed to validate the results of the above GO analyses and to further explore the functional modes of these DEGs in tumors.

We conducted GO analysis on 162 DEGs shared by three gynecological tumors, gaining insight into the biological processes (Figure 5A), cellular components (Figure 5B), and molecular functions (Figure 5C) involved in these genes. To validate the GO analysis results, we further performed GSVA (gene set variation analysis) on these DEGs. The GSVA analysis revealed significant differences between the radiosensitive and radioresistant groups (Figure 5D).

**FIGURE 6:**
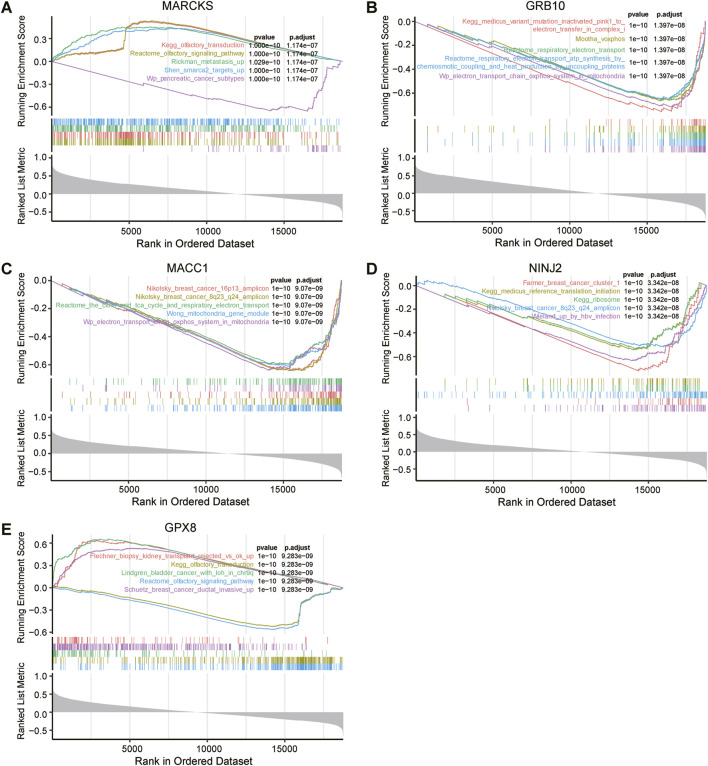
Genomic enrichment analysis (GSEA) of five key genes associated with UCEC prognosis. MARCKS **(A)**, GRB10 **(B)**, MACC1 **(C)**, NINJ2 **(D)**, GPX8 **(E)** were all independently subjected to GSEA to assess their possible functional and biological significance in UCEC prognosis.

To uncover the pathways enriched for prognosis-related genes in the radioresistant group, we performed GSEA (Gene Set Enrichment Analysis) on five key genes related to UCEC prognosis (Figure 6). The results demonstrated enrichment of these genes in specific pathways. The GSEA results also indicated that low expression of the MACC1, GRB10 and MARCKS genes, along with high expression of the NINJ2 gene, were associated with tumourigenesis, including breast, pancreatic and bladder cancers.

### 3.6 Survival analysis and risk factor linkage analysis of key genes

**FIGURE 7:**
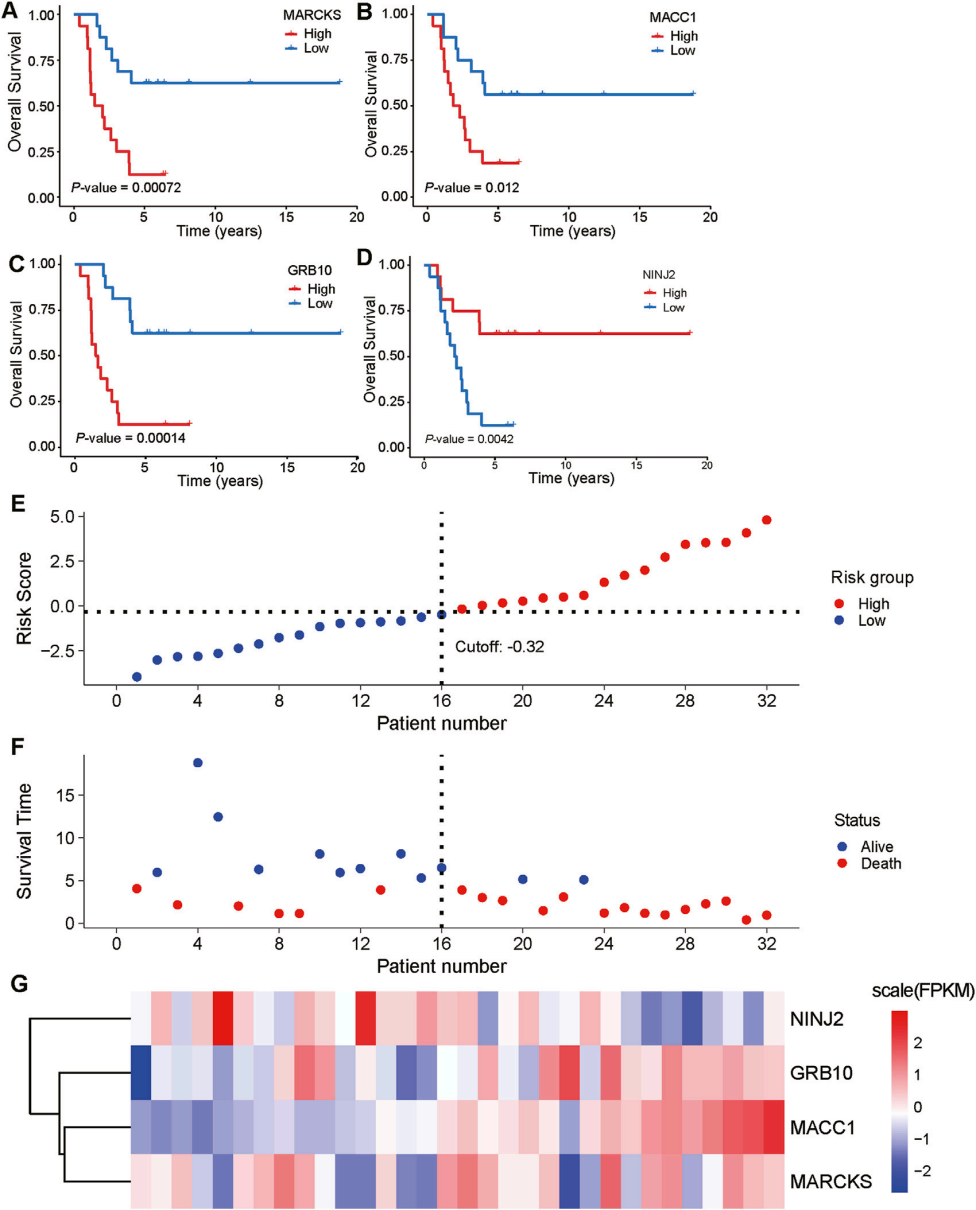
Expression of key prognostic genes in relation to overall survival and risk factors. **(A–D)**: Correlation analyses between the expression levels of five key prognostic genes (MARCKS, MACC1, GRB10, NINJ2, and GPX8) and overall survival are demonstrated. The blue line represents low expression of the genes and the red line represents high expression of the genes. **(E, F)**: A cascade analysis between four genes with significant expression differences (MARCKS, MACC1, GRB10, and NINJ2) and risk factors was performed. **(G)**: Heatmap depicting the expression levels of MARCKS, MACC1, GRB10, and NINJ2 in the low-risk group (first 12 samples) and high-risk group (second 12 samples).

We further explored the relationship between these five key prognostic genes and survival. The analysis showed that low expression of MARCKS, MACC1 and GRB10 was significantly associated with better overall survival, while high expression of NINJ2 was significantly associated with better overall survival (Figures 7A–D). However, no significant correlation was found between GPX8 expression and overall survival. To delve deeper into the discrepancies noted in their survival analyses, we will subsequently focus on these four key genes: MARCKS, MACC1, GRB10, and NINJ2.

To gain a deeper understanding of the relationship between these key genes and risk factors, we performed risk factor linkage analysis on the above key genes with significant expression differences (Figures 7E, F). The results showed that in the high-risk group, the expression levels of MARCKS, MACC1, and GRB10 were elevated, whereas the expression of NINJ2 was decreased (Figure 7G). These findings align with the results of survival analysis, further validating the important role of these genes in predicting the prognosis of UCEC patients.

### 3.7 COX regression forest analysis and column lines to predict the prognosis of UCEC patients

**FIGURE 8:**
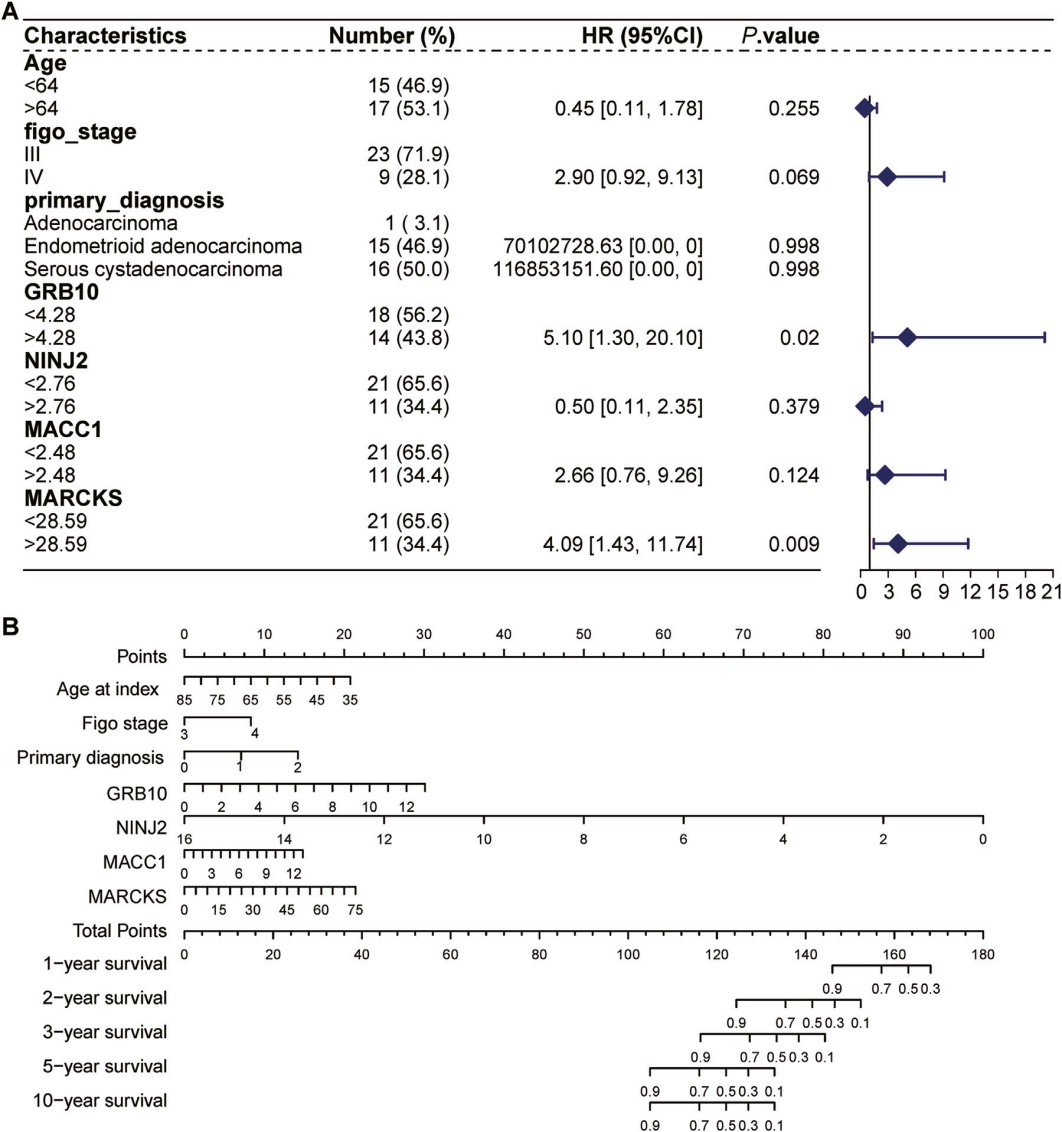
COX regression forest analysis and column lines to predict the prognosis of UCEC patients. **(A)**: COX regression forest analysis based on the TCGA UCEC dataset to assess the impact of age, FIGO stage, initial diagnosis, and four key genes (GRB10, NINJ2, MACC1, MARCKS) on the prognosis of patients with UCEC. **(B)**: The nomogram selects key variables, including age, FIGO staging, initial diagnostic status, and four key genes (GRB10, NINJ2, MACC1, MARCKS). In the figure, each line not only labels the range of values available for each variable, but also cleverly visualizes the magnitude of the variable’s impact on the clinical outcome event through the length of the line segment. Specifically, the influence of each variable at different values is represented by “individual scores” (Points), which are labeled directly on the graph lines, facilitating the understanding of the contribution of each variable when acting independently. Further, the individual scores of all variables are summed to obtain a “Total Point”, which is a composite of the overall impact of all the variables together and corresponds to the corresponding survival percentage.

Using the TCGA UCEC dataset, we performed a COX regression forest analysis to assess the impact of age, FIGO staging, initial diagnosis, and four key genes (GRB10, NINJ2, MACC1, MARCKS) on the prognosis of UCEC patients (Figure 8A). The results showed that the expression of GRB10 and MARCKS significantly affect the patient prognosis. This analysis provided insights into how these factors collectively affect patient survival.

The nomogram, an intuitive and practical tool, aids physicians in more accurately predicting a patient’s prognosis and subsequently tailoring a more personalized treatment plan. We employed the nomogram to comprehensively analyze the aforementioned variables and predict their effects on the survival time of UCEC patients (Figure 8B). The line segments within the bar chart represent the contribution of each factor to the probability of the outcome variable’s occurrence. Specifically, this column line graph facilitates the assessment of radiation tolerance by predicting the distribution of points across various predictors and subsequently calculating a total score. A higher total score is associated with a shorter survival time. For instance, a total score below 104 indicates a 90% probability of surviving more than 5 years, whereas a score exceeding 144 suggests a 90% likelihood of surviving approximately 1 year. Among these factors, the UCEC patients with high expression had poorer survival rates compared with low expression of MARCKS, MACC1, and GRB10, whereas the UCEC patients with high expression had higher survival rates compared with low expression of NINJ2.

### 3.8 Validation of key genes using the CPTAC database

To further confirm the reliability of the genes screened for radiosensitivity and radioresistance in UCEC, we utilized the CPTAC database for review. However, the dataset had a limited number of radioresistant (7 cases) and radiosensitive (6 cases) UCEC samples due to the challenges associated with sample acquisition. Among the four key genes—MARCKS, MACC1, GRB10, and NINJ2—we observed that the expression trends of two, namely, MARCKS and NINJ2, were consistent with our previous analysis results (Supplementary Figure S1A). Although this concordance has not yet reached statistically significance, it still supports our previous findings. Additionally, we performed a survival analysis using this dataset, which showed that the expression patterns of MARCKS and NINJ2 were consistent with those obtained from the TCGA dataset (Supplementary Figures S1B–E). Due to the limited sample size in the CPTAC database, we continue to place greater confidence in the conclusions derived from the TCGA database.

## 4 Discussion

Radiation therapy undoubtedly holds a pivotal role in the treatment of UCEC. However, achieving a comprehensive understanding of radiosensitivity is crucial for enhancing treatment efficacy and optimizing patient outcomes. Regrettably, studies exploring the genetic underpinnings of radiosensitivity based on clinical data remain scant. Consequently, our study aimed to delve deeply into the molecular mechanisms governing radiosensitivity and radioresistance in UCEC samples.

In this study, we initially screened 765 genes from 12 radiosensitive UCEC samples and compared them with 20 radioresistant samples, revealing significant differences between the two groups. The distinct expression patterns of these genes in radiosensitive versus radioresistant samples suggest their potential significance in modulating UCEC radiosensitivity. To pinpoint the key genes influencing UCEC prognosis, we employed Lasso regression and a random survival forest model for rigorous screening. Through meticulous comparison and analysis, we successfully screened five key genes, MARCKS, MACC1, GRB10, NINJ2 and GPX8. Among these, MARCKS plays a crucial role in various cellular activities, including cell adhesion, motility, mucin secretion, cytokinesis, and inflammatory response. Previous studies have implicated MARCKS in aberrant signaling during the development and progression of multiple cancer types, driving cancer metastasis by regulating cancer cell migration and invasion (Chiu et al., 2022). Inhibition of MARCKS has been shown to impair cell proliferation, invasion, migration, and mammosphere formation, and therefore, positioning it as a potential therapeutic target for 28% of MARCKS-positive inflammatory breast cancer patients (Manai et al., 2022). However, whether MARCKS modulates cancer cell migration and invasion in UCEC to impact radiosensitivity requires further investigation. Clarifying its specific role in UCEC radiosensitivity and assessing its potential as a therapeutic target are ongoing research priorities. MACC1 is closely linked to the development, invasion, and metastasis of variety malignancies (Stein et al., 2008). GRB10, meanwhile, is involved in regulating cancer onset and progression, encompassing critical aspects such as cell metabolism, growth, and apoptosis (Ren et al., 2023). Furthermore, NINJ2, a member of the homophilic adhesion molecule family, has recently emerged as a significant player in tumorigenesis and progression (Yan et al., 2023; Zhang et al., 2024). GalR3, a cancer-dependent gene, plays a role in cancer progression (Kiezun et al., 2022), albeit its specific role in endometrial cancer unreported. In contrast, GPX8 shows high expression in most cancers, including endometrial cancer (Zhijing et al., 2022). The key genes we screened are all closely related to cancer development. In UCEC, variations in their expression may influence cancer cell radiosensitization and, consequently, therapeutic efficacy. Notably, despite identifying these genes, their precise mechanisms of action still await further exploration. Additionally, our study has limitations, such as a relatively small sample size and insufficient experimental validation. Future research should aim to expand the sample size, conduct extensive bioinformatics analysis, and perform experimental validation to reveal the exact mechanism of action by which these key genes contribute to UCEC radiosensitization.

In our in-depth analysis of these key genes, we found a positive correlation between them and 24 disease genes intimately linked to UCEC tumorigenesis within the no-response group, reinforcing their significance in UCEC radiation resistance. Additionally, we examined the immune infiltration levels between radiosensitive and radioresistant groups and found a decreased proportion of Macrophages.M0 in the radiation-resistant group. Notably, Macrophages.M0 have been established as a prognostic factor in endometrial adenocarcinoma, positively correlating with favorable disease outcomes (Pan et al., 2021). Our findings align with these reports and offer crucial insights into the intrinsic relationship between tumor microenvironment and radiosensitivity.

To elucidate the specific roles of these key genes in UCEC, we conducted GO and GSVA analyses, revealing their involvement in various biological processes such as cell proliferation, differentiation, apoptosis, and signal transduction. Furthermore, GSEA analyses highlighted their enrichment in specific signaling pathways, providing a foundational understanding of their mechanisms in UCEC.

Survival analysis revealed that low expression of MARCKS, MACC1 and GRB10 was associated with better survival outcomes, whereas high expression of NINJ2 was also linked to favorable survival. This suggests potential biomarker for predicting the prognosis of UCEC patients in clinical settings. Additionally, through COX regression forest analysis and column-line graph prediction, we verified the effects of GRB10, MACC1 and MARCKS on UCEC patient prognosis. Specifically, patients with UCEC exhibiting elevated expression levels of MARCKS, MACC1, and GRB10 experienced reduced survival rates, whereas those with increased expression of NINJ2 showed improved survival outcomes. The study revealed that heightened expression of radioresistant genes (MARCKS, MACC1, GRB10) corresponds with higher prognostic scores and shorter survival durations, whereas higher expression of the radiation-sensitive gene NINJ2 is associated with lower prognostic scores and longer survival periods. These findings propose that these genes may serve as valuable prognostic biomarkers for UCEC.

Regrettably, over the past year or two, we have faced challenges in obtaining tumor tissue samples post-radiotherapy for endometrial cancer due to various reasons. Specifically, the survival status of these patients post-radiotherapy varied widely, making further tissue collection ethically and practically challenging. This limitation impacted our ability to conduct more detailed functional studies and experimental validations.

In conclusion, through comprehensive bioinformatics analysis, this study unveiled the molecular mechanisms underlying radiosensitivity and radioresistance in UCEC, identifying several key genes. These findings enhance our understanding of radiosensitivity in UCEC and suggest new approaches to improve treatment outcomes for UCEC patients in clinical settings. However, this study represents a preliminary exploration, and future work will require additional experimental validation and clinical data to further substantiate these findings and facilitate their application in clinical practice.

## Data Availability

The original contributions presented in the study are included in the article/Supplementary Material, further inquiries can be directed to the corresponding authors.
