# Supplementary material for: Identification of key genes underlying radiosensitivity and radioresistance in endometrial cancer through integrated bioinformatics analysis
Source: Front Genet. 2025 Jan 24;16:1469610. doi: 10.3389/fgene.2025.1469610 (PMC11802559; doi:10.3389/fgene.2025.1469610)
Supplement: Supplementary file 2 [file DataSheet1.DOCX]

Supplemental figure legend:

Supplemental figure 1: Validation of key genes using the CPTAC database. (A) Utilizing the Clinical Proteomic Tumor Analysis Consortium (CPTAC) database, we validated the differential expression of the MARCKS, MACC1, GRB10, and NINJ2 genes in the radiosensitive and radioresistant groups. (B) Correlation analyses between the expression levels of four key prognostic genes (MARCKS, MACC1, GRB10, and NINJ2) and overall survival are presented. The blue line represents low expression of the genes, while the red line represents high expression of the genes.
